# A new species of *Coelotes* Blackwall, 1841 (Araneae, Agelenidae, Coelotinae) from Huaping National Nature Reserve, northeast Guangxi, southern China

**DOI:** 10.3897/BDJ.14.e178386

**Published:** 2026-01-09

**Authors:** Hegui Wang, Runze Jiang, Guchun Zhou

**Affiliations:** 1 School of Life Sciences, National Navel Orange Engineering Research Center, Gannan Normal University, Ganzhou, Jiangxi 341000, China School of Life Sciences, National Navel Orange Engineering Research Center, Gannan Normal University Ganzhou, Jiangxi 341000 China

**Keywords:** Asia, coelotine spiders, distribution, morphology, taxonomy

## Abstract

**Background:**

The genus *Coelotes* Blackwall, 1841, comprises 156 species, primarily distributed in East Asia, including Japan (64 species), China (56 species) and Korea (1 species). Currently, only two species of this genus have been recorded in Guangxi, China.

**New information:**

A new coelotine species, *Coelotes fani* Zhou, **sp. nov.** (♂♀), is described from the Guangxi Zhuang Autonomous Region, China. This includes a detailed description, diagnosis, illustrations and a distribution map of this species.

## Introduction

The genus *Coelotes* was established by Walckenaer (1830). Wang and Jäger (2007) reported 38 species worldwide and divided them into four species groups. Zhu et al. (2017) subsequently recorded 58 species of this genus in China. The main diagnostic characteristics of *Coelotes* can be summarised as follows: (1) the male palp with one patellar apophysis and two tibial apophyses; embolus slender, conductor relatively simple; (2) the female epigyne has a large atrial base with lateral condyle, two epigynal teeth, a slit-like copulatory opening with distinct spermatheca head. In recent years, Chinese arachnologists have revised the genus *Coelotes* in China. Wang and Zhang (2018) transferred five species of *C. pseudoterristris*-group to the genus *Sinocoelotes* Zhao & Li, 2016: *S. porus* (Zhang, Zhu & Wang, 2017), *S. pseudoguangxian* (Wang, Griswold & Ubick, 2009), *S. songi* (Zhang, Zhu & Wang, 2017), *S. yanhengmei* (Wang, Griswold & Ubick, 2009) and *S. yunnanensis* (Schenkel, 1963). Most recently, Luo et al. (2023) transferred two species to the genus *Baiyuerius* Zhao, Li & Li, 2023: *B. globasus* (Wang, Peng & Kim, 1996) and *B. rugosus* (Wang, Peng & Kim, 1996).

The Huaping National Nature Reserve (25°31′–25°36′N, 109°48′–109°58′E) in northeast Guangxi Zhuang Autonomous Region is located in the south-western area of Longsheng County (Wu et al. 2009). Liu et al. (2025) reported a new species of the genus *Nigorella* Wesołowska & Tomasiewicz, 2008 of Salticidae from this Reserve: *N. huaping* Liu & Zhang, 2025. Guangxi Zhuang Autonomous Region located in the southern China, on the south-eastern edge of Yunnan-Guizhou Plateau and west of Guangdong and Guangxi hills. The region has a subtropical monsoon climate. To date, only three *Coelotes* species have been recorded from this area: *C. capacilimbus* Xu & Li, 2006, *C. ningmingensis* Peng et al., 1998, *C. tenutubilaris* Zhang, Zhu & Wang, 2017 (Peng et al. 1998, Xu and Li 2006, Zhu et al. 2017, World Spider Catalog 2025).

## Materials and methods

Specimens were collected using handpicking and sweeping methods, then preserved in 80% ethanol. The female genitalia were dissected and cleared in a trypsin enzyme solution prior to examination and photography. Specimens were examined and measured using a Leica MZ6 stereomicroscope. Photographs were taken with a Kuy Nice CCD mounted on an Olympus BX41 and processed using Helicon Focus software (version 3.10, free) (Khmelik and Kozub 2005). Maps were created using ArcGis 10.2 and subsequently modified with Adobe Photoshop 2021 Extended (Fig. 4). Leg measurements are provided in the following order: total length (femur, patella, tibia, metatarsus, tarsus), with all measurements expressed in millimetres (mm). The specimens are deposited in the Taxidermy Museum of Gannan Normal University, Ganzhou City, China (GNNU). Terminology and taxonomic descriptions follow Zhu et al. (2017) and Kong et al. (2025).

Abbreviations: **A** = atrium; **ALE** = anterior lateral eye; **AME** = anterior median eye; **C** = conductor; **CD** = copulatory duct; **CDA** = dorsal apophysis of conductor; **CF** = cymbial furrow; **CO** = copulatory opening; **E** = embolus; **EB** = embolic base; **ET** = epigynal teeth; **FD** = fertilisation duct; **H** = epigynal hood; **MA** = median apophysis; **MOA** = median ocular area; **MR** = median ridge; **PA** = patellar apophysis; **PLE** = posterior lateral eye; **PME** = posterior median eye; **RTA** = retrolateral tibial apophysis; **S** = spermatheca; **SH** = spermathecal heads; **ST** = subtegulum, **T** = tegulum, **TS** = tegular sclerite.

## Taxon treatments

### Coelotes fani

Zhou sp. nov.

5EE65957-B71F-51E1-AB46-D918D7EEF9C2

1E5F595E-D14C-4CE4-B296-45034919DFA2

#### Materials

- **Type status:** Holotype. **Occurrence:** recordedBy: Fan H.F.; individualCount: 1; sex: male; lifeStage: adult; occurrenceID: C01E2D38-60A1-5DD2-9123-52E431D5C9DD; **Taxon:** kingdom: Animalia; phylum: Arthropoda; class:  Arachnida; order: Araneae; family: Agelenidae; genus: Coelotes; **Location:** country: China; stateProvince: Guangxi Zhuang Autonomous Region; county: Longsheng Autonomous County; locality: Guilin City, Huaping National Nature Reserve; verbatimElevation: 1245; verbatimLatitude: 25.5523439°N; verbatimLongitude: 109.9621197°E; **Event:** samplingProtocol: by hand; year: 2025; month: 11; day: 19; **Record Level:** institutionCode: GXGL-25-165-01
- **Type status:** Paratype. **Occurrence:** recordedBy: Fan H.F.; individualCount: 1; sex: female; lifeStage: adult; occurrenceID: BF33924C-60FC-5651-8234-B81E0BB9E8BE; **Taxon:** kingdom: Animalia; phylum: Arthropoda; class:  Arachnida; order: Araneae; family: Agelenidae; genus: Coelotes; **Location:** country: China; stateProvince: Guangxi Zhuang Autonomous Region; county: Longsheng Autonomous County; locality: Guilin City, Huaping National Nature Reserve; verbatimElevation: 1245; verbatimLatitude: 25.5523439°N; verbatimLongitude: 109.9621197°E; **Event:** samplingProtocol: by hand; year: 2025; month: 11; day: 19; **Record Level:** institutionCode: GXGL-25-165-02
- **Type status:** Paratype. **Occurrence:** recordedBy: Fan H.F.; individualCount: 1; sex: female; lifeStage: adult; occurrenceID: 20681C4E-47E8-570B-907B-BC0FD21932A7; **Taxon:** kingdom: Animalia; phylum: Arthropoda; class:  Arachnida; order: Araneae; family: Agelenidae; genus: Coelotes; **Location:** country: China; stateProvince: Guangxi Zhuang Autonomous Region; county: Longsheng Autonomous County; locality: Guilin City, Huaping National Nature Reserve; verbatimElevation: 1245; verbatimLatitude: 25.5523439°N; verbatimLongitude: 109.9621197°E; **Event:** samplingProtocol: by hand; year: 2025; month: 9; day: 30; **Record Level:** institutionCode: GXGL-25-144-02
- **Type status:** Paratype. **Occurrence:** recordedBy: Zhou G.C.; individualCount: 1; sex: female; lifeStage: adult; occurrenceID: 30A54EA2-66AE-5C5B-BA81-FF65BDEBAB15; **Taxon:** kingdom: Animalia; phylum: Arthropoda; class:  Arachnida; order: Araneae; family: Agelenidae; genus: Coelotes; **Location:** country: China; stateProvince: Guangxi Zhuang Autonomous Region; county: Longsheng Autonomous County; locality: Guilin City, Huaping National Nature Reserve; verbatimElevation: 1251; verbatimLatitude: 25°33'9.446"N; verbatimLongitude: 109°57'39.182"E; **Event:** samplingProtocol: by hand; year: 2025; month: 12; day: 7; **Record Level:** institutionCode: GXGL-25-176-03
- **Type status:** Paratype. **Occurrence:** recordedBy: Zhou G.C.; individualCount: 1; sex: female; lifeStage: adult; occurrenceID: E41A9AEA-2C4F-5FF3-8742-63C3D819CFC5; **Taxon:** kingdom: Animalia; phylum: Arthropoda; class:  Arachnida; order: Araneae; family: Agelenidae; genus: Coelotes; **Location:** country: China; stateProvince: Guangxi Zhuang Autonomous Region; county: Longsheng Autonomous County; locality: Guilin City, Huaping National Nature Reserve; verbatimElevation: 1251; verbatimLatitude: 25°33'9.446"N; verbatimLongitude: 109°57'39.182"E; **Event:** samplingProtocol: by hand; year: 2025; month: 12; day: 7; **Record Level:** institutionCode: GXGL-25-176-04

#### Description

**Figure 1.**
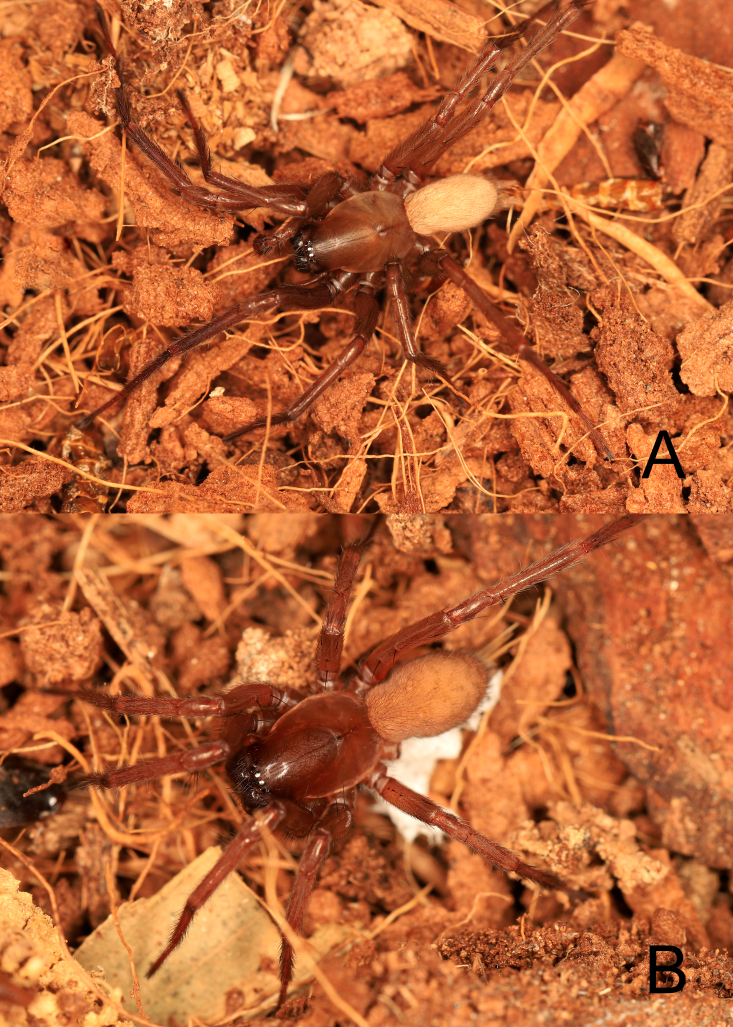
Living photos of *Coelotes fani* Zhou, sp. nov. **A** male (holotype); **B** female (paratype).

**Figure 2.**
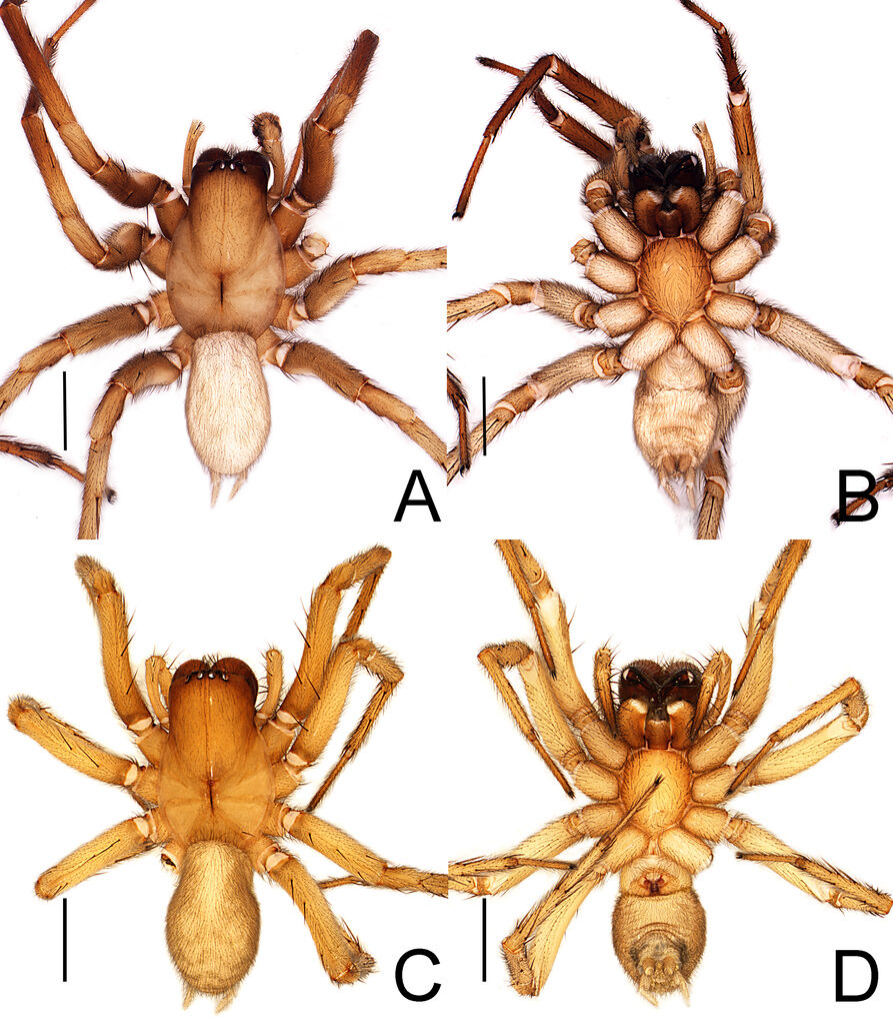
*Coelotes fani* Zhou, sp. nov., habitus. **A, B** male (holotype); **C, D** female (paratype). A, C dorsal view; B, D ventral view. Scale bars: 2 mm (A-D).

**Male** (holotype, GXGL-25-166-01): Habitus as in Fig. 1A and Fig. 2A, B. Total length 7.68. Carapace 4.32 long, 2.91 wide. Eye (Fig. 2A) sizes and interdistances: AME 0.11, ALE 0.18, PME 0.17, PLE 0.18; AME–AME 0.06, AME–ALE 0.05, PME–PME 0.11, PME–PLE 0.11, ALE–PLE 0.06. MOA: 0.35 long; 0.29 front width, 0.41 back width. Clypeus height 0.26. Chelicerae with five promarginal and five or six retromarginal teeth. Leg measurements: I 13.13 (3.68, 1.35, 3.11, 3.12, 1.87); II 11.72 (3.32, 1.23, 2.52, 2.97, 1.68); III 9.61 (3.03, 0.77, 1.87, 2.68, 1.26), IV 15.67 (4.06, 1.42, 3.48, 4.52, 2.19). Leg formula: 4123. Opisthosoma 3.48 long, 2.96 wide, yellowish-brown and body surface with pale grey stripes, visible on the dorsal surface.

**Figure 3.**
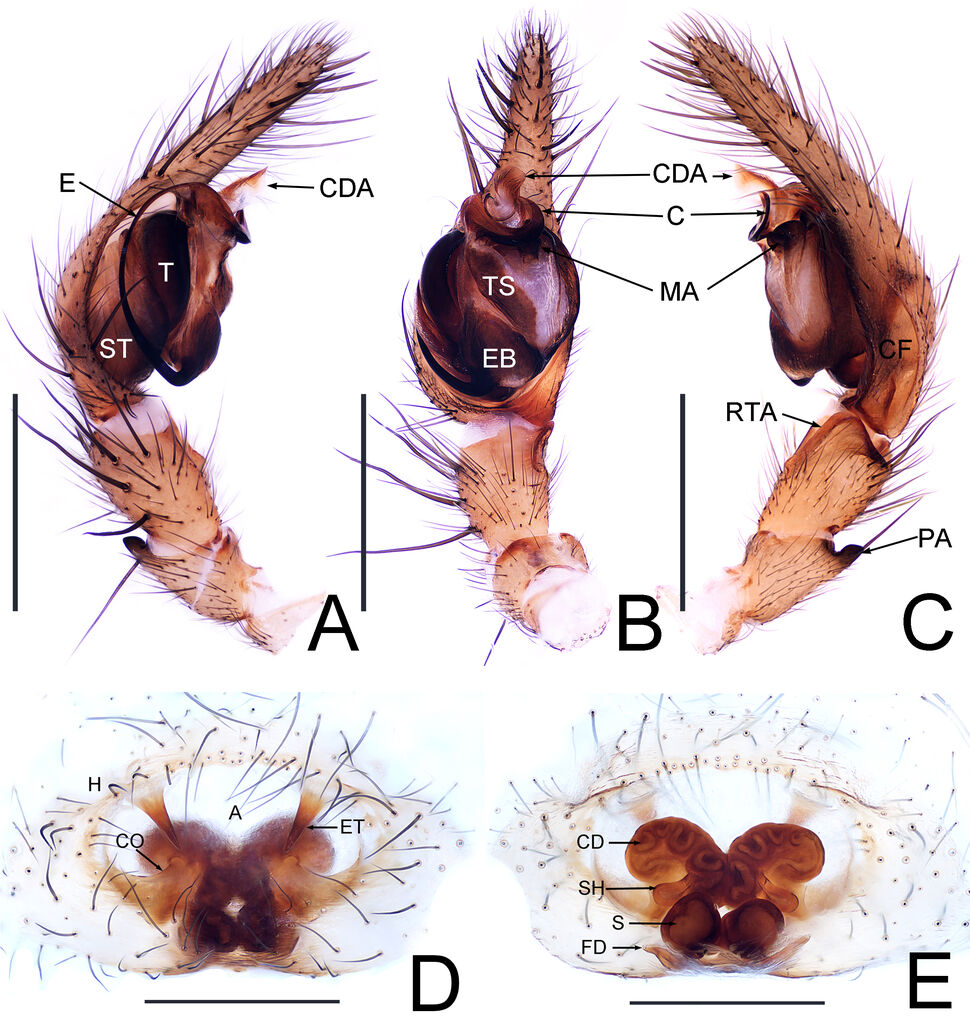
*Coelotes fani* Zhou, sp. nov.: **A** left palp, prolateral view; **B** same, ventral view; **C** same, retrolateral view; **D** epigyne, ventral view; **E** vulva, dorsal view. Abbreviations: A = atrium, C = conductor, CD = copulatory duct, CO = copulatory opening, E = embolus, EB = embolic base, ET = epigynal teeth, FD = fertilisation duct, H = epigynal hood, H = epigynal hood, MA = median apophysis, PA = patellar apophysis, S = spermatheca, SH = spermathecal heads, ST = subtegulum, T = tegulum, TS = tegular sclerite, RTA = retrolateral tibial apophysis. Scale bars: 0.5 mm (A-E).

**Palp** (Fig. 3A–C). Patella dorsally with thick long spine; patellar apophysis (PA) somewhat rectangular, present retrolaterally, apically with small outgrowth (Fig. 3C). Lateral tibial apophysis (LTA) thumb-shaped with blunt end; retrolateral tibial apophysis (RTA) apex somewhat triangular, about two-thirds of tibial length (Fig. 3C). Cymbial furrow (CF) shallow, about one-third of cymbium, bottom end blunt (Fig. 3C). Median apophysis (MA) ear lobe-shaped (Fig. 3B); conductor (C) concave, ventrally grooved, base course, apical end curved inwards (Fig. 3A); embolic base (EB) wide and thick, with obvious separation line (Fig. 3B); embolus (E) whip-shaped (Fig. 3B).

**Female** (paratype, GXGL-25-144-02): Habitus as in Fig. 1B and Fig. 2C and D. Total length 7.88. Carapace 4.21 long, 2.82 wide. Eye (Fig. 2C) sizes and interdistances: AME 0.12, ALE 0.18, PME 0.18, PLE 0.18; AME–AME 0.06, AME–ALE 0.08, PME–PME 0.12, PME–PLE 0.17, ALE–PLE 0.04. MOA: 0.33 long; 0.25 front width, 0.43 back width. Clypeus height 0.24. Chelicerae with five promarginal and six retromarginal teeth. Leg measurements: I 11.35 (3.41, 1.33, 2.47, 2.53, 1.61); II 9.71 (2.73, 1.72, 2.01, 2.27, 1.42); III 9.02 (2.47, 1.21, 1.87, 2.27, 1.21), IV 12.84 (3.87, 1.33, 2.87, 3.41, 1.37). Leg formula: 4123. Opisthosoma length 3.67, width 2.27, abdomen tawny, back fine hairy, markings lighter.

**Epigyne** (Fig. 3D and E). Atrium (A) distinct and wide; epigynal tooth (ET) spiky, present anteriorly; copulatory opening (CO) convoluted, present in the middle with nest-shaped depression (Fig. 3). Copulatory duct (CD) convoluted, present anteriorly; spermathecal head (SH) thick, drop-shaped, points to a 45° angle (Fig. 3E); spermatheca (S) subcircular; fertilisation duct (FD) conspicuous, horn-like, extending laterally (Fig. 3E).

**Variation**: Total length in females (n = 4) varies from 7.88 to 10.65.

#### Diagnosis

The new species resembles *Coelotes mastrucatus* Wang, Yin, Peng & Xie, 1990 (Zhu et al. (2017): figs. 83A-E) in having the similar abdomen pattern in the both sexes. The male can be distinguished by: (1) lateral tibial apophysis (LTA) half the length of the tibia (Fig. 3B), vs. 2/3 of the length of the tibia (Zhu et al. (2017): fig. 83E); (2) conductor (C) bends to the back and hook-shaped (Fig. 3C), vs. conductor first bends backward and then upwards and and blunt end (Zhu et al. (2017): fig. 83E); (3) dorsal apophysis of conductor (CDA) bends upwards and fan-shaped (Fig. 3B) vs. CDA inwardly curved and obtusely pointed end (Zhu et al. (2017): fig. 83D). The female can be distinguished by: (1) epigynal tooth (ET) point to the inner edge of copulatory opening (Fig. 3D) vs. pointing to copulatory opening (Zhu et al. (2017): fig. 83A); (2) spermathecal head (SH) drop-shaped and the length not exceeding spermatheca (S) (Fig. 3E) vs. SH small and located above the spermatheca (Zhu et al. (2017): fig. 83B).

#### Etymology

The specific name is dedicated to Haofei Fan for his contribution to the collection of specimens; it is a noun in the genitive case.

#### Distribution

**Figure 4.**
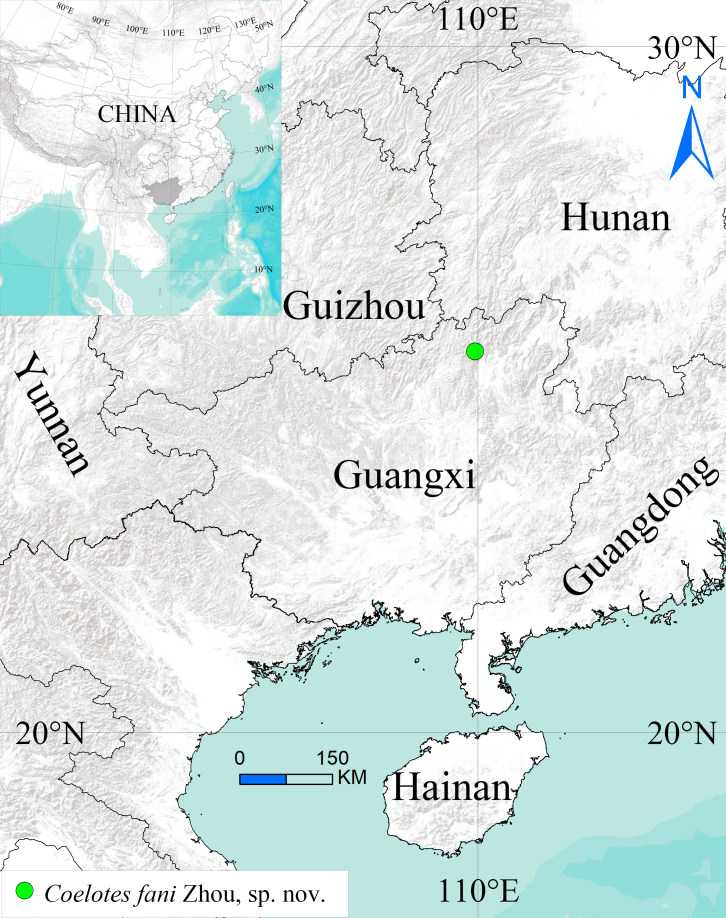
Distribution records of *Coelotes fani* Zhou, sp. nov. from Guangxi Zhuang Autonomous Region, China.

Known only from the type locality in Guangxi Zhuang Autonomous Region, China (Fig. 4).

#### Biology

This species mainly lives under rocks in evergreen broad-leaved forests and frequently searches for prey around cave openings.

## Supplementary Material

XML Treatment for Coelotes fani
